# Mr. Hill, on Cow-Pox

**Published:** 1801-02

**Authors:** G. N. Hill

**Affiliations:** Chester


### *To the Editors of the Medical and Phyſical Journal.*

GENTLEMEN,

CONVINCED that no preliminary Diſſertation is neceſſary to invite your attention, or that of the whole medical world, to any circumſtance which will tend to promote the very general and humane attempts to introduce the Cow-pox: I ſhall preſume to make an obſervation or two, which I venture to believe will tend in ſome degree to promote the grand, benevolent deſign. In the practice of medicine, generally conſidered, it is impoſſible that practitioners can ever devote too much attention to all the minor parts of the ſubject which calls for their conſideration; a want of this care has too frequently defeated the beſt laid plans, and the almoſt apparent certainty of ſucceſs: This obſervation applies more particularly to the attempts at introducing a new diſeaſe; every little circumſtance attached to it is of the higheſt importance, and every endeavour muſt be uſed to combat general as well as local prejudices, to render its good effects as manifeſt as poſſible. The common practice of conveying the variolous matter from one town or village to another, when Inoculation for the Small-pox became pretty general, was by means of threads imbued with the matter, encloſed in paper; a pretty free inciſion was made in each arm to receive the thread, in order that infection might be certain: But when it was found that Small-pox matter was of a very virulent nature, and that portions of it, when detached in this way from the body of a ſufferer, retained their poiſonous nature for a conſiderable time, Operators contented themſelves with barely moiſtening the point of a lancet or needle with it, for uſe on ſome diſtant day; a method which but rarely failed, where every other circumſtance was favorable for for the reception of the difeafe, and attention was paid to the exact point of time when the matter was fitteft for being ufed. Not fo the Cow-pox matter, I apprehend; I am convinced more attention is neceffary on this head, than has yet been given to the fubject. The difference in degree of malignity, or ftrength of the two fluids, feems now fufficiently obvious, and it will be inftantly allowed, that we cannot too forcibly imprefs this truth on the minds of all who will give us a candid hearing. When this point is obtained, and any friend defires to fubmit their child to the operation, it is one of the firft wifhes of both parties, that the difeafe may fucceed after the firft time of inferting the matter: When this defirable event fails to take place, the difappointment is great; and if it occurs often, will tend much to retard the progrefs of the highly important bufinefs.

The firft child I was defired to inoculate with Vaccine matter, refided in the fame houfe with an intelligent Phyfician of this city, who took great pains to procure proper recent matter; it was taken on a lancet in the ufual way, and the day following I ufed it, making a very free fcratch in each arm, fo as to draw blood; which mixing with the gummy matter of the pox, formed a kind of fcab on each little fore: This remained on fome days, but produced no infection. At the end of fixteen or eighteen days, a frefh fupply of matter being obtained, I made the incifions larger, carefully moiftened the dried fluid with fteam, &c.; but to as little purpofe as before. Notwithftanding thefe difappointments, and the fubject being a delicate child under fix weeks old, the mother cheerfully requefting a third trial, my patient was carried to a houfe where two children were under the difeafe, and inoculated with limpid matter warm from the arm of one of them; this fucceeded, and the difeafe run its ordinary fafe courfe: In the interim I received matter from London and Gloucefterfhire, on thread, inclofed in paper, neither of which, though ufed with every care, produced any effect. As before remarked, confiderable chagrin follows the failure of infection from the firft operation, even in Small-pox; fome parents I have known, who would have deemed it prefumptuous to permit even a fecond trial. The part of the country I live in, although, perhaps, the firft milk county in the kingdom, affords little or no opportunities of collecting the original Cow-pox virus; we have, therefore, hitherto had to procure it from a diftance. To prevent others experiencing the mortification of want of fuccefs the firft time of operating, is my intention in propofing the method of taking and preferving the variolous virus, taught me by my firft preceptor, the honoured and ingenious Mr. James Wyke

 furgeon, furgeon, of Colebrook-dale, to be adopted for the more effectual prefervation of the vaccine.

Procure a fmall phial, the fmaller the better; fit it with a tight cork, having previoufly expelled the damp air from it by placing it near the fire a fhort time; take a thread of cotton yarn, about an inch, or inch and a half long, make a puncture in the vefícle, prefs the centre, or middle part of the cotton when held like a loop, thereon, at the fame time untwifting it a little, thus imbibing all the moifture you can from the part; twift the ends together, and place in your bottle, fo as not to touch its bottom, the cork of which will keep it tightly againft the neck; put it into a warm pocket, and carry it about with you until wanted; when required to be fent to a diftance, though a bottle be not a convenient article to tranfmit by letter, yet we have now the comfort of fuch a number of other conveyances, that this obftacle finks into nothing, when compared with the fatisfaction arifing from fuccefs. When about to be ufed, if the phial be immerfed in hot water a fhort time before uncorking, the matter will rarely be found dry and brittle, as it almoft always is on a lancet; and if it fhould be fo in a fmall degree, ftill it will contain the active principle unevaporated, whilft that on the inftrument is too often wholly inert. Since writing the above, I perceive by a York Newfpaper, Dr. Cappe mentions the advantage refulting from keeping the vaccine matter on glafs, and that from this fubftance it can fuffer no injurious change. Though this be an undoubted fact, as to the matter of glafs itfelf, yet, I apprehend it will not fecure the fluid from the influence of the air, which I imagine will be allowed to be the efficient caufe of injuring its activity in any other way than inclofure in a phial. I need not dwell a moment on the property cotton feems to have of imbibing and retaining contagious miafma. Mr. Simpfon of Malton, Yorkshire, recommends a flip of glafs with the matter *dried* on it, to be run through a cork, and then inclofed in a fuitable bottle; this mode comes neareft the one propofed, but I imagine the cotton will be found preferable, on account of its *not* fuffering the matter to dry fo fpeedily as glafs. Having written thus far, your laft Journal came to hand; I find therein a letter from Dr. Marfhall, at Gibraltar, to Mr. Ring, near the conclufion of which, he fays, "Some of the matter we ufed for inoculation here, was what you obligingly furnifhed us with, and we find it perfectly efficacious, although no precautions had been ufed as to the preferving it, more than putting it in a *fmall phial*.

Again, Dr. Waterhoufe, of Cambridge, New England, writes thanks to his friend in Bath for the *phial* of vaccine matter, matter, and ſays, "its activity I have experienced on my neareſt connections:" I have no doubt the learned Profeſſor was well pleaſed at his ſucceſs, but I have every reaſon to believe, had he received an infected lancet, or bit of thread in paper, he would, on the other hand, have been much diſappointed. Before concluding my letter, permit me to add an obſervation on the means of inducing the poorer claſſes of the community to avail themſelves of this invaluable gift of Providence, for, unleſs we can cauſe *them* to liſten to our plan, unleſs we can fully arreſt *their* attention, our progreſs, I fear, will be but ſlow; on the contrary, this deſirable object once attained, the buſineſs will be complete. All the medical gentlemen of this city have zealouſly come forward with a tender of their ſer-ſervices to inoculate the poor, gratis, whenever applied to for that purpoſe; this is a great ſtep, but ſomething more muſt be done; their underſtandings muſt be convinced of the ſafe, ſalutary, and certain conſequences of the operation, before they will apply to us to ſerve them. Would not the printing, on a ſuitable paper, a few of the cleareſt, moſt ſtriking facts, in the plaineſt language, and freely circulating them amongſt individuals of this deſcription, joined with the powerful force of example in the higher orders, have this effect? I am, &c.

*Cheſter, Nov.* 17, 1800. G. N. HILL.

